# Corrigendum

**DOI:** 10.1002/jcla.24324

**Published:** 2022-04-08

In Volume 36, issue 1, 2022, in the article “CircSamd4: A novel biomarker for predicting vascular calcification” by Yuting Zhou, Yehong Liu, Shiyi Xuan, Tianhui Jin, Ke Chen, Zufei Wu, Wentao Su, Liang Chen, Gangjun Zong, the article title was incorrect.

The correct title is CircSamd4a: A novel biomarker for predicting vascular calcification.

